# Odorant Receptors and Odorant-Binding Proteins as Insect Pest Control Targets: A Comparative Analysis

**DOI:** 10.3389/fphys.2018.01163

**Published:** 2018-08-24

**Authors:** Herbert Venthur, Jing-Jiang Zhou

**Affiliations:** ^1^Laboratorio de Química Ecológica, Departamento de Ciencias Químicas y Recursos Naturales, Universidad de La Frontera, Temuco, Chile; ^2^Center of Excellence in Biotechnology Research Applied to the Environment (CIBAMA), Universidad de La Frontera, Temuco, Chile; ^3^Department of Biological Chemistry and Crop Protection, Rothamsted Research, Harpenden, United Kingdom; ^4^Jilin Provincial Key Laboratory of Animal Resource Conservation and Utilization, Northeast Normal University, Changchun, China

**Keywords:** insect olfaction, modulators, antagonists, agonists, pest management, odorant binding, chemosensory receptors

## Abstract

Recently, two alternative targets in insect periphery nerve system have been explored for environmentally-friendly approaches in insect pest management, namely odorant-binding proteins (OBPs) and odorant receptors (ORs). Located in insect antennae, OBPs are thought to be involved in the transport of odorants to ORs for the specific signal transduction of behaviorally active odorants. There is rich information on OBP binding affinity and molecular docking to bioactive compounds as well as ample 3D crystal structures due to feasible production of recombinant proteins. Although these provide excellent opportunities for them to be considered as pest control targets and a tool to design pest control agents, the debates on their binding specificity represent an obstacle. On the other hand, ORs have recently been functionally characterized with increasing evidence for their specificity, sensitivity and functional roles in pest behaviors. However, a major barrier to use ORs for semiochemical discovery is the lack of 3D crystal structures. Thus, OBPs and ORs have not been analyzed comparatively together so far for their feasibility as pest control targets. Here, we summarize the state of OBPs and ORs research in terms of its application in insect pest management. We discuss the suitability of both proteins as pest control targets and their selection toward the discovery of new potent semiochemicals. We argue that both proteins represent promising targets for pest control and can be used to identify new super-ligands likely present in nature and with reduced risk of resistance development than insect pesticides currently used in agriculture. We discuss that with the massive identification of OBPs through RNA-seq and improved binding affinity measurements, these proteins could be reconsidered as suitable targets for semiochemical discovery.

## Introduction

The human population has increased dramatically and is predicted to reach 9 billion in 2050. Food crops must be cultivated and managed to meet their demand and to increase their resistance against the damage by insect pests and crop diseases. Such situations have been managed mainly by artificial chemicals, such as insecticides and fungicides, whose persistence has caused food contamination, environmental, and health concerns, and called for alternative and integrated pest management strategies to reduce the use of these chemicals. Current specific examples are the concerns on the insecticide neonicotinoids for their role in decreasing honey bee populations (Godfray et al., 2014), the resistance to a wide range of insecticides that the peach potato aphid, *Myzus persicae*, has acquired because of intensive insecticide applications (Bass et al., 2014) and the spreading through globalization of several deadly diseases that are transmitted by mosquitoes, such as malaria, yellow fever, dengue and Zika (Jones et al., 2008). Therefore, the use of environmentally friendly approaches has become an attractive strategy to manage insect pests through the identification of behaviorally active chemicals (i.e., semiochemicals) to target insect olfaction systems and to either manipulate insect pest behaviors away from food crops or interrupt their sexual behaviors (Zhou, 2010; Pickett, 2014). Recently, two alternative targets in insect periphery nerve system have been explored for environmentally-friendly approaches in insect pest management, namely odorant-binding proteins (OBPs) and odorant receptors (ORs). Although chemosensory proteins (CSPs) have been also identified and reported to bind odorants (Iovinella et al., 2013; Li H. L. et al., 2016; Peng et al., 2017), their diverse tissue expression and attributed function as well as limited structural studies (e.g., only 5 crystal/NMR structures solved), have made them less attractive as targets.

Insect OBPs have been shown to increase the sensitivity of ORs to odorants using the *Xenopus* oocyte heterologous expression systems and voltage-clamp technique (Syed et al., 2006; Sun et al., 2013b; Zhang Q. H. et al., 2017) and HEK293 cell expression system and Ca-imaging (Grosse-Wilde et al., 2006). So far, a repertoire of OBPs have been identified in small hair-like structures (i.e., sensilla) projected at the surface of antennae from a wide range of insect species and considered as carriers to interact with semiochemicals during peripheral signal transduction (Vogt and Riddiford, 1981; Klein, 1987; Maida et al., 1993; Zhou, 2010; Pelosi et al., 2014). The functional roles of OBP57d and OBP57e of *Drosophila sechellia* and *Drosophila melanogaster* in the host selection have been demonstrated (Matsuo et al., 2007). Alternative to heterologous expression and binding assays for OBPs, the authors knocked out the expression of OBP57d and OBP57e in *D*. melanogaster and demonstrated that both proteins are important for the unique food preference of *D. sechellia*. Furthermore, when OBP57d/e genes were introduced from *D. sechellia* to *D. melanogaster*, the oviposition behavior of *D. melanogaster* shifted to the *D. sechellia*'s host plant *Morinda citrifolia*. Therefore, OBPs have been explored as targets for semiochemical discovery. For instance, the OBP1 of the mosquito *Culex quinquefasciatus* CquiOBP1 was used to identify an attractive blend comprising trimethylamine (TMA) and nonanal by using gas chromatography-electroantennographic detection (GC-EAD) and *in vitro* binding assays along with field bioassays (Leal et al., 2008). Likewise, the identification of a potent attractant (methyl eugenol) of the fruit fly *Bactrocera dorsalis* was performed based on a general odorant-binding protein (GOBP) BdorGOBP (Jayanthi et al., 2014). In this study, the authors identified methyl eugenol by protein structure prediction, molecular docking and dynamics along with tryptophan fluorescence quenching assay followed by behavioral bioassays of 25 chemicals. More recently, the OBP7 of the parasitoid wasp *Sclerodermus* sp. (SspOBP7) was used to screen behaviorally active chemicals (Yi et al., 2018). From a group of 19 chemicals, only 6 were found to bind to SspOBP7 in the fluorescence quenching binding assays. Subsequent behavioral olfactometry bioassays confirmed that *Sclerodermus* sp. showed significant preference to only 2 compounds, (+)-α-longipinene and terpinolene that had a good binding affinity with SspOBP7. This so called reverse chemical ecology approach has accelerated the understanding of olfactory mechanisms and the discovery of active chemicals that could be used to manipulate insect behaviors for pest management (Leal, 2017). Since then, the discovery of ORs have further provided more sensitive targets for such reverse chemical ecology (Wetzel et al., 2001; Sakurai et al., 2004; Corcoran et al., 2014; Wang et al., 2016). These ORs act as the secondary filter for olfactory information and molecular recognition in insect antennae, converting chemical signals to electrical impulses that provoke behavioral responses (Kaissling, 2013; Bohbot and Pitts, 2015). The number of ORs varies across insect species from around 40 candidates in Lepidopterans, such as the codling moth *Cydia pomonella* (Bengtsson et al., 2012), the oriental leafworm moth *Spodoptera litura* (Feng et al., 2015) and the tobacco hawk moth *Manduca sexta* (Grosse-Wilde et al., 2011), to more than 70 candidates in the African malaria mosquito *Anopheles gambiae* (Rinker et al., 2013) and the pea aphid *Acyrthosiphon pisum* (Richards et al., 2010), and 170 candidates annotated in the honey bee *Apis mellifera* genome (Robertson and Wanner, 2006). These insect receptors function only with a highly conserved and co-expressed co-receptor (ORco) as heteromeric transmembrane complexes heterologous expression systems. This is completely different from those of other animal G-protein coupled receptors (Civelli et al., 2013), which provides unique opportunities for the development of insect pest specific control agents.

Although functional and structural characteristics as well as biotechnological applications of insect OBPs have been widely reviewed (Zhou, 2010; Leal, 2013; Pelosi et al., 2014), a comparative analysis of ORs and OBPs in terms of their use as targets for semiochemical discovery has not been made so far. On one hand, OBPs highlight as extracellular soluble proteins with significant experimental 3D structure information available and straightforward protocols for semiochemical screening by means of fluorescence binding characterizations. However, their broad specificity, wide distribution in non-olfactory tissues and secondary functions (e.g., scavengers, solubilizers, and regeneration) make their selection as targets a difficult task for semiochemical discovery. Similarly, ORs are transmembrane proteins with no experimental 3D structure information available so far and more sophisticated to be expressed and purified in heterologous systems. However, most of the studied ORs show high sensitivity to very specific chemical groups. Some insect ORs have been successfully used as targets to identify new semiochemicals, such as *C. quinquefasciatus* OR36 (Choo et al., 2018). It is also possible to use them in the identification of antagonists that could serve as a new approach to disrupt the behavior of a given insect pest (Chen and Luetje, 2012, 2013, 2014). Therefore, the objective of this review is to summarize the suitability and application of both ORs and OBPs in view of the discovery of pest control agents and discuss their further perspectives in insect pest management strategies.

## Insect odorant-binding proteins: functional and structural features toward pest control agent discovery

The first insect OBP was identified more than 30 years ago by Vogt and Riddiford (1981). Currently, a large number of OBPs, particularly pheromone-binding proteins (PBPs) and general odorant-binding protein (GOBP) in Lepidopterans, has been identified across insect species with more than 2000 amino acid sequences of insect OBPs deposited so far in NCBI database (https://www.ncbi.nlm.nih.gov) and classified into subgroups based on the number of highly conserved cysteine residues: classic, minus-C, plus-C, and atypical (Zhou et al., 2004; Venthur et al., 2014) after initial classification of PBP, GOBP, and antennal specific protein (ASP). Moreover, recent analyses of insect antennal transcriptomes have shown that insects express several OBPs highly in their antennae (Table 1). Despite the identification of ample insect OBPs, most of the functional studies have been relied on the OBPs expression profiles in insect antennae as well as their activity-structure binding relationships determined by means of fluorescent competitive binding and molecular docking.

**Table 1:** Number of insect OBPs and ORs identified from antennal or head transcriptome studies based on RNAseq data.

**Insect species**	**Insect order**	**OBPs**	**ORs**	**References**
*Manduca sexta*	Lepidoptera	18	47	Grosse-Wilde et al., 2011
*Cydia pomonella*	Lepidoptera	–	43	Bengtsson et al., 2012
*Dendrolimus houi*	Lepidoptera	23	33	Zhang et al., 2014
*D. kikuchii*	Lepidoptera	27	33	Zhang et al., 2014
*Spodoptera litura*	Lepidoptera	21	26	Feng et al., 2015
*Spodoptera exigua*	Lepidoptera	45	51	Du et al., 2018
*Chilo suppressalis*	Lepidoptera	26	47	Cao et al., 2014
*Agrotis ipsilon*	Lepidoptera	22	35	Gu et al., 2014
*Grapholia molesta*	Lepidoptera	28	48	Li G. et al., 2015
*Ostrinia furnacalis*	Lepidoptera	23	56	Zhang T. et al., 2015
*Athetis dissimilis*	Lepidoptera	–	60	Dong et al., 2016
*Conogethes punctiferalis*	Lepidoptera	15	46	Jia et al., 2016
*Helicoverpa armigera*	Lepidoptera	34	60	Zhang J. et al., 2015
*H. assulta*	Lepidoptera	29	64	Zhang J. et al., 2015
*Hedya nubiferana*	Lepidoptera	–	49	Gonzalez et al., 2017
*C. fagiglandana*	Lepidoptera	–	49	Gonzalez et al., 2017
*C. nigricana*	Lepidoptera	–	48	Gonzalez et al., 2017
*Mythimna separata*	Lepidoptera	32	71	Chang X. Q. et al., 2017
*Eogystia hippophaecolus*	Lepidoptera	29	63	Hu et al., 2016a
*Oraesia emarginata*	Lepidoptera	41	35	Feng et al., 2017
*Plodia interpunctella*	Lepidoptera	29	47	Jia et al., 2018
*Hyphantria cunea*	Lepidoptera	30	52	Zhang et al., 2016
*Lobesia botrana*	Lepidoptera	35	61	Rojas et al., 2018
*Chouioia cunea*	Hymenoptera	25	80	Zhao Y. et al., 2016
*Apis cerana cerana*	Hymenoptera	17	74	Zhao H. et al., 2016
*Aenasius bambawalei*	Hymenoptera	54	226	Nie et al., 2018
*Osmia cornuta*	Hymenoptera	6	48	Yin et al., 2013
*Bemisia tabaci*	Homoptera	8	–	Wang R. et al., 2017
*Adelphocoris suturalis*	Hemiptera	16	–	Cui et al., 2017
*Nilaparvata lugens*	Hemiptera	10	–	Zhou S. S. et al., 2014
*Sitobion avenae*	Hemiptera	13	–	Xue et al., 2016
*Halyomorpha halys*	Hemiptera	30	–	Paula et al., 2016
*Phenacoccus solenopsis*	Hemiptera	12	4	Nie et al., 2018
*Empoasca onukii*	Hemiptera	40	–	Bian et al., 2018
*Tropidothorax elegans*	Hemiptera	19	–	Song et al., 2018
*Calliphora stygia*	Diptera	28	50	Leitch et al., 2015
*Anopheles gambiae*	Diptera	79	75	Rinker et al., 2013
*Drosophila melanogaster*	Diptera	50	61	Shiao et al., 2013
*Episyrphus balteatus*	Diptera	49	51	Wang B. et al., 2017
*Eupeodes corollae*	Diptera	44	42	Wang B. et al., 2017
*Bradysia odoriphaga*	Diptera	49	–	Zhao et al., 2018
*Dendroctonus valens*	Coleoptera	21	22	Gu et al., 2015
*Anomala corpulenta*	Coleoptera	24	93	Chen et al., 2014
*Anoplophora glabripennis*	Coleoptera	42	37	Hu et al., 2016b
*Anoplophora chinensis*	Coleoptera	–	53	Sun et al., 2018
*Callosobruchus chinensis*	Coleoptera	21	–	Zhang Y. N. et al., 2017
*Rynchophorus ferrugineus*	Coleoptera	38	76	Antony et al., 2016
*Cylas formicarius*	Coleoptera	33	54	Bin et al., 2017
*Leptinotarsa decemlineata*	Coleoptera	26	37	Liu Y. et al., 2015
*Tenebrio molitor*	Coleoptera	19	20	Liu S. et al., 2015
*Colaphellus bowringi*	Coleoptera	26	43	Li X. M. et al., 2015
*Tomicus yunnanensis*	Coleoptera	45	8	Liu et al., 2018
*Schistocerca gregaria*	Orthoptera	-	119	Pregitzer et al., 2017
*Blattella germanica*	Blatodea	48	5	Niu et al., 2016

### Specificity of insect OBPs

Recent debate around the ligand binding specificity of OBPs has caused concerns for their suitability as targets for semiochemical discovery, with some authors reporting a broad binding capacity to several volatiles (Campanacci et al., 2001; Zhou et al., 2004; Zhou, 2010; Pelosi et al., 2014), while others supporting the remarkable specificity of some OBPs (Qiao et al., 2009; Damberger et al., 2013; Li et al., 2013, 2017). Traditionally, these studies have been performed by the competitive binding assays based on fluorescence displacement (Campanacci et al., 2001). In this competitive displacement binding assay, a fluorescent probe, commonly *N*-phenyl-1-napthylamine (1-NPN), is used for initial binding with OBPs, which is then displaced by the ligands of interest. Therefore, ligands with a high affinity are those with the strong ability to displace 1-NPN from OBP binding pockets at low concentrations of dissociation constants (K_D_) and inhibitory concentrations (IC_50_) assuming the protein is 100% active and the binding stoichiometry is 1:1.

These studies propose some OBPs as specific for chemical properties of compounds. Particularly, the sub-classes OBPs, such as PBPs and GOBPs of Lepidopteran, have shown high specificity for volatile compounds with either particular hydrocarbon lengths or specific functional groups, such as aldehydes, alcohols or esters (Zhou, 2010). For example, it was found that among 16 tested compounds, three compounds with 12 carbon (C12) atoms [codlemone, 1-dodecanol and (*E*,*E*)-2,4-dodecadienal] showed higher affinities to the PBP1 of the codling moth *C. pomonella* (CpomPBP1) with binding affinity constants K_D_ between 2.73 and 5.90 μM (Tian and Zhang, 2016). The GOBP2 of *Bombyx mori* had higher affinity to the sex pheromone bombykol than to its isomer bombykal (Zhou et al., 2009). Likewise, the GOBP1 and GOBP2 of *S. litura* had stronger binding to C14-C16 alcohol-pheromone analogs, such as (*Z*)-9-tetradecanol, (*Z*)-9-hexadecanol, (*Z*)-11-hexadecanol, and (*E*)-11-hexadecanol in fluorescence binding assays and molecular modeling (Liu N. Y. et al., 2015). On the other hand, the presence of phenolic groups in chemicals such as eugenol, isoeugenol, and 4-vinylguaiacol showed to play a key role for the high affinity of the OBP14 in the honeybee *A. mellifera* (Schwaighofer et al., 2014).

OBPs have also been used to screen a large number of chemicals. For instance, nonanal, acetophenone, 6-methyl-5-hepten-2-one and some terpenoids from 41 host odorants showed high binding affinities (11–16 μM of K_D_) to the OBP6 of the alfalfa plant bug *Adelphocoris lineolatus* (AlinOBP6) through fluorescence competitive binding assays (Sun et al., 2017). Interestingly, AlinOBP6 exhibited a good binding affinity to nonvolatile compounds, such as quercetin, gossypol, rutin hydrated, and (–)-catechin, suggesting a broad specificity of AlinOBP6 and likely a role in mechanisms to respond to volatile and non-volatile compounds. Similarly, the binding of 45 volatile organic compounds to the OBP8, OBP9 and OBP10 of the endoparasitoid *Microplitis mediator* was tested by the competitive binding assays (Li et al., 2014). Their findings suggested that nonane, nonanal, farnesol, β-ionone, nerolidol, acetic ether, and farnesene have a high binding affinity to the OBPs in the μM range. Later in behavioral bioassays, β-ionone, nonanal, and farnesene showed attractant activity while nonane and farnesol showed repellent activity. This study supports the role of insect OBPs as targets to discover new semiochemicals that can act as either attractants or repellents, and to screen for super-ligands whether with their native or chemically optimized chemical structure (Hooper et al., 2009). More recently, the screening of host odorants and/or sex pheromones using the fluorescence-based binding assay has been reported for other OBPs with much better binding affinities, though the best binding affinity of the ligands are still in μM ranges. For example, multiples OBPs of the oriental fruit moth *Grapholita molesta* have been studied, showing high affinity of (*E*)-8-dodecadienyl acetate (K_D_ of 2.18 μM) to OBP8, 11 and 15, (*Z*)-8-dodecenyl acetate (K_D_ of 1.09 μM) to PBP1 and PBP2, and dodecanol (K_D_ of 5.10 μM) to OBP4, 5 and 10 (Li G. W. et al., 2016; Chen et al., 2018; Zhang et al., 2018). Similarly, the OBP1 of the scarab beetle *Hylamorpha elegans* suggested β-ionone as the best ligand with a K_D_ value of 6.9 μM among 29 tested host odorants (Venthur et al., 2016). The OBP13 of Japanese pine sawyer *Monochamus alternatus* showed butylated hydroxytoluene as the best ligand with K_D_ of 0.77 μM in 20 tested host odorants (Li et al., 2017), and farnesene was highlighted as the best ligand with a remarkable K_D_ of 0.86 μM for the OBPm2 of the white-striped longhorn beetle *Batocera horsfieldi* among 58 host odorants (Zheng et al., 2016).

Thus, comprehensive studies on insect OBPs using the competitive binding assays have reported specific groups of high affinity ligands in the μM range from a broad list of candidates. The OBP binding studies so far face a major challenge to measure the binding affinity from μM to nM range, which is normally regarded as high affinity binding in other biokinetic studies. However, it has been well-established for semiochemical discovery by these binding studies using OBPs that: (1) Reproducible protocols are available to clone, express, purify and test binding specificity of insect OBPs and (2) Fluorescence-based binding assays provide a robust technique to perform the rapid experimental screening for a relatively large number of chemicals. However, recent findings for the OBP1 of *Aenasius bambawalei* (AbamOBP1) report the binding of the protein with lower K_D_ at acid pHs, inconsistent with the better binding at basic pHs in previous studies. The authors report that the binding stoichiometry between AbamOBP1 and tested ligands was not 1:1, which is likely caused by the presence of dimers or even trimers of OBPs and, therefore, a 100% active protein could not be assumed, suggesting false positives from the competitive binding assays (Li et al., 2018). It appears that a combined methodology such as fluorescence intrinsic quenching assays (Bette and Breer, 2002) could be in better accordance with behavioral assays. An example of dimeric forms of OBPs have been reported by Wang et al. (2013), where mixtures of recombinant OBP1 and 2 of the scarab beetle *Holotrichia oblita* as well as OBP2 and 4 of the same insect, showed higher binding affinities to odorants, such as β-ionone and retinol, than the OBPs alone. Later, the authors revealed that such OBP pairs were actually co-localized each in the same sensilla by immunocytochemical analyses.

### Structural features of insect OBPs

The heterologous expression of insect OBPs in bacteria and the subsequent three-dimensional (3D) structure determination by either X-ray crystallography or NMR or the prediction by homology modeling provide substantial information and have attracted a great interest recently for OBPs as a suitable target for pest management strategies. The research of insect OBPs has started to focus on structural characterizations since Sandler et al. (2000) reported the first crystal structure of the PBP1 of *B. mori* and its interactions with the sex pheromone, bombykol [(*E*,*Z*)-10,12-hexadecadien-1-ol]. There are about 70 X-ray crystal and 5 NMR protein structures solved and deposited in Protein Data Bank database (https://www.rcsb.org/pdb/home/home.do) to date due to their small molecular weight and ease to be expressed and purified. Most of the structures are related to OBPs of *A. gambiae, D. melanogaster, B. mori*, and *A. mellifera* with 24, 12, 10, and 9 structures, respectively. The Classic OBPs are characterized by 6 α-helices connected by 3 disulfide bridges in a specific motif pattern C1-X_25−30_-C2-X_3_-C3-X_36−42_-C4-X_8−14_-C5-X_8_-C6 (Xu et al., 2003), being the most studied and reviewed OBP subfamily so far (Zhou et al., 2004; Pelosi et al., 2006, 2014; Venthur et al., 2014; Brito et al., 2016). However, the identification of OBPs from other non-Lepidopteran insects relieved that such sequence motif patterns can vary and have been further grouped as minus-C OBP subfamily with 4 cysteines residues (Hekmat-Scafe et al., 2002; Weinstock et al., 2006), plus-C OBP subfamily with 3 extra cysteines and a conserved proline (Zhou et al., 2004) and atypical OBP subfamily with more cysteines in C-terminal section (Xu et al., 2003). On the other hand, the diversity and non-homologous feature of OBPs among insect genera could serve as advantages in the development of semiochemicals or even insecticides for specific insect species.

Indeed, molecular modeling approaches, such as homology modeling, have allowed, in most cases, an extensive study of their structural characteristics in complement with *in vitro* binding assays (Venthur et al., 2014). This computer-based method (i.e., *in silico*) is an approach of using experimental 3D structures as templates to predict the 3D structure of a target protein based only on its amino acid sequence (Leach, 2001; Schmidt et al., 2014). Early structural studies of insect OBPs were limited by the availability of a few crystal structures and the low percentage of sequence identity to known OBP structures (e.g., < 30%). Thus, probable 3D arrangements of OBPs with no further refinement were reported (Campanacci et al., 2001; Ban et al., 2003; Tsuchihara et al., 2005; Paramasivan et al., 2007). These studies have served as a good starting point. However, with more X-ray crystal and NMR structures available for different insect orders, this *in silico* complementary protein/ligand interaction research has become more comprehensive and routine with methods such as dynamics simulations and molecular docking. For example, the ligand-binding mechanisms of minus-C OBP21 of *Dastarcus helophoroides* (DhelOBP21) were studied first by homology modeling and molecular docking and then supported by fluorescence binding assays (Li D. Z. et al., 2015). The authors proposed that hydrophobic interactions between ligands and DhelOBP21 are more crucial for binding than hydrogen bonds, and molecules with a size of 100-125 Å^3^ are the most suitable. More recently, the structural approaches based on extensive dynamics simulations (110 ns) of DhelOBP21-ligand complexes [(+)-β-pinene, camphor and β-caryophyllene] have shown the remarkable conformational stability of DhelOBP21/(+)-β-pinene complex which strongly supports the behavioral activity of *D. helophoroides* (Yang et al., 2017). Similarly, Tian et al. (2016) explored the structural features of the PBP2 of *C. pomonella* and demonstrated that hydrophobic and hydrogen bond interactions as well as chain length of C12 atoms and the unsaturation of compounds are key features during ligand binding. Likewise, the 3D structure prediction for the OBP of *B. horsfieldi* (BhorOBPm2) helped to demonstrate that long chain (C14) compounds had higher affinities than those with shorter chains due to the flexibility of its binding pocket (Zheng et al., 2016). The conformational flexibility of OBPs for odorant binding has also been reported for the minus-C OBP14 of *A. mellifera* (Schwaighofer et al., 2014). Interestingly, the authors compared the wild-type OBP14 with a mutant version of the OBP14 in which a third disulfide bridge was added and evaluated their thermal stability when they bound to volatiles. Their findings showed that a constricted flexibility in the mutant OBP14 resulted in its lower binding affinities than the wildtype OBP to some volatiles, such as eugenol, methyl eugenol, isoeugenol, and other phenolic-based compounds.

The structural studies in OBPs have allowed more specific research into the mechanisms of odorant binding and release in order to predict the OBP/ligand interactions in the olfactory system of insects. This could further advance in using OBPs as targets and a tool to design pest control agents. It has been reported that insect OBPs display an outstanding pH-dependent mechanism of odorant binding and release, which certainly contributes to the specific properties of these proteins. This process supports the idea that these proteins are able to bind odorants at a basic pH (6.5), transport and release them at an acid pH (4.5) (Lautenschlager et al., 2005; Xu et al., 2011; di Luccio et al., 2013). It is proposed from the structure studies that the odorant molecule is ejected to ORs because the long C-terminal section displaces it from the OBP binding pocket when the C-terminal section shifts at acid pHs from an extended structure to a helical form and inserts inside the binding site. The pH-dependent approach helped to elucidate the selective role of a PBP in *B. mori* (BmorPBP). Thus, Damberger et al. (2013), through the study of pH-dependent polymorphism of BmorPBP by NMR, reported that this protein is able to eject bombykol near the OR at an acid pH, whereas ligands with low binding affinity are released before they reach the vicinity of receptors. The pH-independent structures are also observed in the OBP1 of *Locusta migratoria* (LmigOBP1) (Zheng et al., 2015). However, the studies on some OBPs have proposed different mechanisms. For instance, although the long C-terminal tail in the GOBP2 of *B. mori* also forms an α-helix, it is located across the N-terminal helix and not buried into the binding site as in BmorPBP1 (Zhou et al., 2009). Likewise, the OBP13 of *M. alternatus* (MaltOBP13) exhibits high binding capacity at acid pH (5.0) than at basic pH (7.4) for several ligands, especially α-terpinolene, with a K_D_ of 56.93 μM at pH 7.4 and 7.20 μM at pH 5.0 (Li et al., 2017).

The identification of semiochemicals, 3D structure prediction of OBPs, their binding mechanisms and the characterization of specificity determinants have provided an outstanding opportunity to use insect OBPs as targets in pest control management. Furthermore, the introduction of other structure-based methodologies, such as quantitative structure-activity relationship (QSAR) (Oliferenko et al., 2013), will enhance OBP's roles as targets for semiochemical discovery and optimization.

## Insect odorant receptors as promising pest control targets

**Figure 1:**
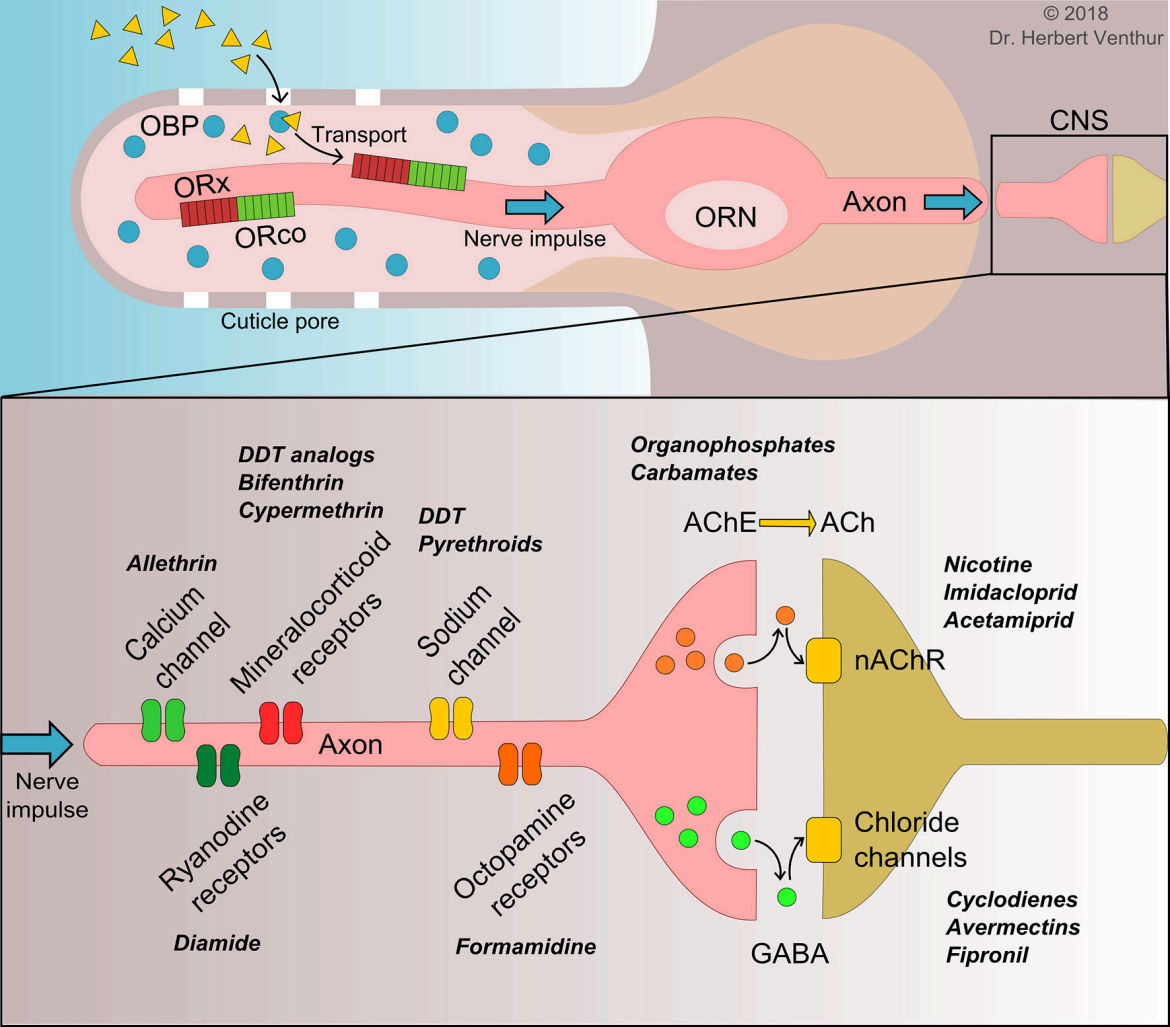
Actions of semiochemicals on olfactory receptor neuron (ORN) in periphery nerve system and mode of action of pesticides in central nervous system (CNS). The top panel represents the current understanding actions of semiochemical (yellow triangles) from entrance into insect sensilla through cuticle pores to their binding and transporting by odorant binding proteins (OBPs) to insect unique olfactory receptor complex (ORx/ORco). The bottom panel represents the mode of action of different classes of pesticides in the CNS along with the targeted receptors and enzymes reported crucial for pest resistance development to a particular type of insecticides (bold cursive). Small orange and green circles indicate neurotransmitters. GABA, γ-aminobutyric acid; AChE, acetylcholinesterase; Ach, acetylcholine; nAChR, nicotinic acetylcholine receptor.

Insect ORs are another important component of the periphery nerve system and a key player in the signaling transduction pathway in the antennae for insect behaviors, which begins with OBP binding to ligands, transporting to ORs, and terminating by degrading enzymes which are thought to remove the ligands away from the neuron dendrite of ORs (Leal, 2013). Insect ORs are G protein-coupled receptors (GPCRs) with seven transmembrane domains. Hopf et al. (2015) suggested the 3D structure of *D. melanogaster* OR85b and ORco using a coevolution homology modeling approach. Their findings indicate a structural arrangement based on seven TMHs and a C-terminal faced to the extracellular section and N-terminal to the intracellular section (Figure 1) unlike the GPCRs of other animals (Tsitoura et al., 2010), being a different topology from those of animal GPCRs (Katritch et al., 2013). This unique feature of insect ORs from animal GPCRs places them as ideal insect specific targets to be deployed for pest control management.

Currently, a range of 40–80 *ORs* have being identified based on antennal transcriptome data from insect species, such as moths, beetles, flies, and mosquitoes (Table 1) with even more than 500 *ORs* based on genome sequencing in ants (Table 2). Uniquely, despite the divergence of insect *ORs*, there is a highly conserved *OR* (Larsson et al., 2004; Jones et al., 2005) across insect species, originally identified as *OR83b* and later renamed as *ORco* (Vosshall and Hansson, 2011). It forms a functional heteromeric complex with other ORs (ORx/ORco) (Neuhaus et al., 2005). This gives another dimension for insect OR to be consider as pest control targets in addition to their unique topology. However, the complex of insect ORs with ORco *in vivo* in the dendrite of olfactory receptor neurons (ORNs) are less reported. The ORco subunit has received special attention due to its high conservation across insect species, from structural features such as several motifs in its C-terminal section (Ray et al., 2014) to its ancestry presence before the appearance of other ORx (Missbach et al., 2014). Particularly, the TMH6 of ORco has been proposed as a pore domain that plays a role in the regulation of cation flow (Wicher et al., 2008; Carraher et al., 2015). In the same direction, the function of ORco has been elucidated by the use of RNA-interference (RNAi) that knocks down the expression of *ORco* genes. For instance, when the *ORco* expression was knockdown in the gypsy moth *Lymantria dispar*, its antennal electrophysiological response (electroantennographic response or EAG) to the sex pheromone (i.e., disparlure) was significantly decreased from 1.472 to 0.636 mV (Lin et al., 2015). Similarly, RNAi was used to reduce the expression of *ORco* gene in the true bug *Apolygus lucorum*, resulting in a decrease of EAG responses to two semiochemicals, (*E*)-2-hexenal and (*E*)-2-hexenyl butyrate (Zhou Y. L. et al., 2014). More recently, *ORco* knockdown by RNAi negatively affected the oviposition and blood ingestion in the Chagas disease vector *Rhodnius prolixus*, which was later confirmed through a series of bioassays (Franco et al., 2016). These scientific evidence is consistent with the proposal that ORco forms metabotropic gated cation channels which controls threshold responses to odorants (Stengl and Funk, 2013). Likewise, RNAi approach supports the role of either a specific OR/ORco complex or ORco alone in the recognition of agonists. Hence, making them suitable targets for identifying or optimizing novel molecules with semiochemical activity in insect pest management (Taylor et al., 2012; Tsitoura and Iatrou, 2016).

**Table 2:** Number of insect OBPs and ORs identified from insect genome studies.

**Insect species**	**Insect order**	**OBPs**	**ORs**	**References**
*Bombyx mori*	Lepidoptera	–	64	Xia et al., 2008
*Drosophila melanogaster*	Diptera	50	60	Clark et al., 2007
*Camponotus floridanus*	Hymenoptera	–	139	Bonasio et al., 2010
*Harpegnathos saltator*	Hymenoptera	–	105	Bonasio et al., 2010
*Apis mellifera*	Hymenoptera	21	170	Weinstock et al., 2006
*Tribolium castaneum*	Coleoptera	47	265	Richards et al., 2008
*Anopheles* related species	Diptera	–	60	Neafsey et al., 2015
*Plutella xylostella*	Lepidoptera	38	87	You et al., 2013
*Nasonia* species	Hymenoptera	90	64	Werren et al., 2010
*Danaus plexippus*	Lepidoptera	32	64	Zhan et al., 2011; Vogt et al., 2015
*Culex quinquefasciatus*	Diptera	–	180	Arensburger et al., 2010
*Cerapachys biroi*	Hymenoptera	15	506	Oxley et al., 2014
*Glossina morsitans*	Diptera	32	46	Watanabe et al., 2014
*Aedes aegypti*	Diptera	70	56	Sinkins, 2007
*Acyrthosiphon pisum*	Hemiptera	15	79	Richards et al., 2010
*Rhodnius prolixus*	Hemiptera	27	106	Mesquita et al., 2016
*Linepithema humile*	Hymenoptera	12	367	Smith et al., 2011
*Solenopsis invicta*	Hymenoptera	12	400	Wurm et al., 2011
*Aedes albopictus*	Diptera	86	158	Xu et al., 2016
*Blattella germanica*	Blatodea	109	134	Robertson et al., 2018
*Bemisia tabaci*	Homoptera	8	–	Zeng et al., 2018

### Functional role of insect ORs

The early elegant studies of expressing *B. mori* OR1 (BmorOR1) together with ORco (OR83b) in *Xenopus laevis* oocytes (Sakurai et al., 2004) and HEK293T cells (Grosse-Wilde et al., 2006; Wicher et al., 2008) demonstrated that these receptors function as ligand-gated cation channels that can unleash the influx of extracellular Ca^2+^ in ORNs (Sato et al., 2008). It has been proposed that ORs have an intracellular binding site for calmodulin (Carraher et al., 2015; Bahk and Jones, 2016), a ubiquitous protein in eukaryotes that modulates the function of target proteins via intracellular Ca^2+^ signaling. Some structural domains sensitive to odorants, such as the extracellular loop 2 (ECL2) and the transmembrane helices 4 (TMH4), have been demonstrated through the mutations of several amino acids, particularly alanine 195 (Ala195) (Hughes et al., 2014). For instance, it was shown that the sensitivity of *A. gambiae* OR15 (AgamOR15) to acetophenone was significantly decreased when Ala195 was mutated to isoleucine. Rahman and Luetje (2017) confirmed that the key role of Ala195 in AgamOR15 is to function as a part of an inhibitor interaction site. Another study (Leary et al., 2012) showed that Ala148 in the OR3 of the moth *Ostrinia nubilalis* (OnubOR3) alters the response to a specific pheromone when it was mutated to threonine, decreasing ~14-fold the sensitivity to (*E*)-11-tetradecenyl acetate, hence, selectively narrowing the specificity of OnubOR3.

More recently, the functional role of 17 ORs in *Spodoptera littoralis* toward 51 chemicals emitted by flowering plants was deorphanized using a high-throughput approach based on cloning and expression of the ORs in *Drosophila* fly embryos followed by single-sensillum recordings (SSRs) (De Fouchier et al., 2017). The authors propose that some receptors that recognize aromatic compounds have emerged first and are more conserved, whereas receptors tuned to terpenes and aliphatic compounds (e.g., sex pheromones) have emerged more recently and evolved faster. In the same context, a specific expansion of ORs for floral odorants has been reported for generalist honey bees (e.g., *A. mellifera and A. cerana*), which is not present in specialist bees, such as *Dufourea novaeanglicae* and *Habropoda laboriosa* (Karpe et al., 2017). Thus, it seems that the range of host plants for a given insect leads to the divergence of *ORs*, showing the olfactory process as a constantly evolving system and specific. This would be a useful point for the design of pest control agents for a given insect species, and the evidence of a reduced possibility of developing resistance to these agents by the insect pests.

Besides the structural and functional characteristics of ORs, its divergence represents a putative guide of the different odor sources for what the insect olfactory system is tuned for. Supporting the above, a remarkable difference in ORs between the fruit fly *D. melanogaster* and the mosquito *A. gambiae* has been reported. An important specie-specific *OR* divergence has been detected so that more than 20 *A. gambiae OR*-related genes have no homologous partners in *D. melanogaster*, and around 18 *D. melanogaster ORs* have no corresponding genes in *A. gambiae* (Hill et al., 2002). Similarly, a specific expansion of 175 *OR* genes was identified through genome and phylogenetic analyses in the honey bee *A. mellifera*, which is potentially explained by their broad olfactory perception to chemicals, such as diverse pheromone blends and floral odors (Robertson and Wanner, 2006). On the contrary, less expansions are reported in the bedbug *Cimex lectularius* with only 48 genes encoding *ORs*, which is likely explained by its limited host range as blood feeder (Benoit et al., 2016). This dynamic evolutionary process for *ORs* provides other aspects in semiochemical identification, especially for those insect pests with a wide range of hosts.

### Agonist identification using ORx subunit

The functional study of ORs along with the conserved ORco has provided exquisite evidence of their role in perceiving chemicals regarded as agonists. It has been suggested that ORs could act as generalist or specialist for semiochemicals (Bohbot and Dickens, 2012). Thus, non-pheromonal compounds could be recognized by generalist ORs and pheromones are only recognized by specialist ORs (Hughes et al., 2010). For example, ORs from the noctuid moths *S. littoralis* and *S. litura* have been extensively studied in terms of their sensitivity to volatile agonists. Thus, the OR6 of *S. littoralis* (SlitOR6) was expressed in *Drosophila* olfactory neurons and found to specifically recognize a pheromone component of *S. littoralis*, (*Z*,*E*)-9,12-tetradecenyl acetate (Montagné et al., 2012). Similarly, OR13 and OR16 of *Spodoptera exigua* (SexiOR13 and SexiOR16) were functionally characterized against pheromone components. It was shown that SexiOR13 was highly sensitive to (*Z*,*E*)-9,12-tetradecenyl acetate and (*Z*)-9-tetradecenyl acetate, whereas SexiOR16 was even more sensitive to (*Z*)-9-tetradecenyl acetate only (Liu et al., 2013). More research has been published with the functional study of candidate pheromone receptors (PRs), such as the OR1 of *B. mori* (Syed et al., 2006) or *Plutella xylostella, Mythimna separate*, and *Diaphania indica* (Mitsuno et al., 2008; Liu Y. et al., 2018); the OR6, OR13, OR14, OR15 and OR16 of *Heliothis virescens* (Wang et al., 2011); the OR1, OR3, and OR6a of *C. pomonella* (Cattaneo et al., 2017) and the OR1 of *M. sexta* (Wicher et al., 2017). All these studies are carried out by selecting candidate PRs based on phylogenetic analysis and/or male specific expression. In the meantime, alternative approaches for precise PR selection might seem difficult due to a large number of ORs and variable expressions. For instance, are ORs differentially expressed when insects are faced to certain conditions such as the exposure to sex pheromones or virgin/mated? This has recently been probed for the *OR3, OR6*, and *OR11* of *S. exigua*, where these genes were differentially expressed when the insects were exposed to synthetic pheromone (Wan et al., 2015). The authors reported more than 1- to 3-fold increase in the relative expression after the exposure. On the other hand, age and mating status seem not to affect the expression of ORs. It was found that the *OR13* and *OR15* of both *H. virescens* and *H. subflexa* are mainly expressed in males, and stable in terms of their relative expression for virgin males of 2 h, 1, 2, 4, and 8 d old as well as 4-day old mated males (Soques et al., 2010). Finally, it is worth mentioning that apart from PRs of Lepidopterans, the functional roles of non-pheromone receptors (non-PRs) have also been addressed. The specificity of BmorOR1 to bombykol was probed by Sakurai et al. (2004) through the heterologous expression of the OR1 in *Xenopus* oocytes and demonstrated the corresponding ORco as the essential unit for the function of the OR1. This allowed the functional study of other receptors, not only of Lepidopterans, but also of insect species from different orders, such as aphids, mosquitoes and beetles. An example is the OR12 of *S. litura* (SlitOR12), which was expressed in *Xenopus* oocytes and its sensitivity to odorants was tested by electrophysiology. Their results indicate that SlitOR12 is highly sensitive to (*Z*)-3-hexenyl acetate, a common green leaf volatile (GLV), suggesting a key role during oviposition and/or host location by females (Zhang et al., 2013). An OR from aphid *A. pisum* (ApisOR4) was functionally characterized through expression in *Xenopus* oocytes and electrophysiology (Zhang R. B. et al., 2017). Their findings suggest a specificity of ApisOR4 to 8 volatiles that belong to aromatic and terpenoid class. Similarly, the high sensitivity of *A. pisum* OR5 (ApisOR5) to the alarm pheromone, (*E*)-β-farnesene, was elucidated by Zhang R. et al. (2017) and further corroborated when the ApisOR5 was knocked down by RNAi treatments, resulting in *A. pisum* individuals not repelled by (*E*)-β-farnesene. The OR7, OR10, and OR88 of the mosquito *Aedes albopictus* were tested in terms of odor recognition (Liu et al., 2016). The authors report the OR10 and OR88 are highly sensitive to human-derived odorants, such as indole and 1-octen-3-ol. Contrary to what was expected, the mosquitoes that were treated with RNAi to significantly depress the OR10 and OR88 expressions were still able to respond to indole and 1-octen-3-ol. This may imply that other generalist ORs likely complement the lack of specialist receptors for host seeking behavior. More recently, the reverse chemical ecology approach has been reported based on the responses to 230 odorants by the OR36 of *C. quinquefasciatus* expressing in *Xenopus* oocytes, resulting in acetaldehyde as not only the strongest agonist, but also behaviorally active as oviposition attractant in bioassays (Choo et al., 2018). The specificity of 17 ORs from *S. littoralis* to low concentrations of ligands (pM range) (De Fouchier et al., 2017) has been demonstrated. Interestingly, some SlitORs (OR14, OR24, OR15, OR27, and OR29) even seemed to be sensitive at less than 1 pmol of ligand flux when SSRs were performed.

Although volatile compounds with agonist activity have been screened against ORs, a specific chemical with strong agonist effect on mosquitoes, 2-(4-Ethyl-5-(pyridin-3-yl)-4*H*-1,2,4-triazol-3-ylthio)-*N*-(4-ethylphenyl)acetamide (VUAA1), has opened the field of research (Jones et al., 2011; Taylor et al., 2012). Later studies by Taylor et al. (2012) provided evidence of VUAA1-derived chemicals, such as VUAA4, able to increase its agonist effect by 10-fold on ORco from *A. gambiae, H. virescens*, and *Harpegnathos saltator*. Interestingly, the authors reported that any change on amide substituents will cause a complete loss of agonist activity. This yields helpful insights into the structural requirements of agonists and the structure-activity relationship between VUAA analogs and ORs. Finally, despite the enhanced agonist activity of VUAA chemicals, its relatively high molecular weight (367.47 g mol^−1^ for VUAA1) vs. volatile agonists, such as bombykol (238.42 g mol^−1^), makes a direct volatile delivery of VUAA something not feasible. With that in mind, the searching for smaller structural analogs represents an interesting focus of research.

### Antagonism onto ORco subunit

Along with the study of VUAA-related analogs that can act as strong agonists, the blockage of ORco by antagonists has also emerged to guide semiochemicals and pesticide design. Thus, a structural analog of VUAA1, VU0183254 (2-(4-Ethyl-5-furan-2-yl-4H-[1,2,4]triazol-3-ylsulfanyl)-1-phenothiazin-10-yl-ethanone), was reported to inhibit ORco response, acting as allosteric modulator in *A. gambiae* and disrupting the recognition of agonists such as eugenol by the complex OR65/ORco (Jones et al., 2012). Other VUAA-structural analogs have also been reported as antagonists. An example is the N-,2-substituted triazolothioacetamide compounds OLC3 and OLC12 that disrupts the ORco response in a similar fashion in *C. quinquefasciatus, A. gambiae, D. melanogaster*, and *O. nubilalis*, suggesting a conserved binding site in ORco (Chen and Luetje, 2012). Considering the inhibition of ORco as a promising strategy to disrupt behaviors of insects, it seems that subsequent efforts should aim at the compounds with lower molecular weight than VUAA-derived antagonists. For example, OX1a (232 g mol^−1^), tryptamine (160.22 g mol^−1^) and isopropyl cinnamate (190.24 g mol^−1^) were reported to have antagonist effect on ORco (Chen and Luetje, 2013, 2014; Tsitoura et al., 2015) with roughly half or less molecular weight than VUAA1. Nevertheless, future use of these antagonists should be studied carefully, since the blockage of the conserved *ORco* can affect not only harmful insects, but also beneficial ones.

Besides the antagonist effect probed *in vitro*, the evidence at behavioral level supports the idea that structural analogs of pheromones can function as antagonists. For example, Sellanes et al. (2010) reported the inhibition of sexual response in the honeydew moth *Cryptoblabes gnidiella* when the structural analogs, (*Z*)-9-tetradecenyl formate and (*Z*)-11-hexadecenyl formate, were added to synthetic sex pheromone, (*Z*)-11-hexadecenal and (*Z*)-13-octadecenal, in wind tunnel tests. This pheromone antagonist effect was later corroborated in field assays, where the trapping of *C. gnidiella* males decreased in a dose-dependent pattern. The pheromone antagonism has also been reported for *B. mandarina*, an ancestor of *B. mori* (Daimon et al., 2012). Their findings corroborate bombykol as the sex pheromone, and bombykal [(*E*,*Z*)-10,12-hexadecadienal] and bombykyl acetate [(*E*,*Z*)-10,12-hexadecadienyl acetate] as antagonists, which strongly inhibited the attraction of males in field to the sex pheromone bombykol. More recently, evidence of pheromone antagonism was reported for the snout moth *Herpetogramma submarginale*. When (*Z*)-13-hexadecenol was added to its sex pheromone, (*Z*)-13-hexadecenyl acetate, significantly decreased the number of males captured in field (Yan et al., 2015). The pheromone antagonism seems based on the differences in chemical functional group such as alcohols, aldehydes and esters depending on the insect species. Nevertheless, the antagonist effect of these structural analogs might not be due to ORco inhibition but the specificity of ORx to antagonists. A recent study suggests that the OR16 of *Helicoverpa armigera* is able to specifically recognize the pheromone antagonist, (*Z*)-11-hexadecenol (Chang H. et al., 2017). The authors supported the specific role of OR16 considering that *H. armigera* females emit the antagonist compound along with its sex pheromone ((*Z*)-11-hexadecenal and (*Z*)-9-hexadecenal) as a strategy to avoid non-optimal mating with immature males. Outstandingly, when the *OR16* was knocked down by the genome editing technique CRISPR/Cas9 and *H. armigera* males were tested by electrophysiology and behavioral assays, no EAG response was recorded and males tried to mate with immature females.

## Odorant receptors vs. binding proteins: pros and cons for insect pest management

For the case of OBPs, the ligand specificity and mechanisms of OBPs represent controversial aspects, which seems strongly dependent on the methods used for the measurement of ligand affinity. For instance, it has been reported that PBPs, such as those from the moths' *P. xylostella* and *Eogystia hippophaecolus*, can bind both sex pheromone components and analogs (Sun et al., 2013a; Hu et al., 2018). This suggests that downstream players such as ORs could enhance specificity and sensitivity of odorant reception. Recent evidence supports that the co-expression of PBPs and PRs can increase the sensitivity toward pheromones. For example, multiple combinations from PR1-4 and PBP1-4 were used to test their response to sex pheromone components of the moth *Chilo suppressalis* (Chang et al., 2015). The authors found a significant increase in sensitivity of response toward (*Z*)-11-hexadecenal when PR4 and PR6 were co-expressed with PBP4. Although the interaction of these proteins could arise a new level of research as pest control targets, the different pairing of PRs and PBPs shed lights on the complexity of the olfactory system in insects, making the approach a difficult task for a large set of compounds and proteins to test. Despite the above, insect OBPs are of small molecular size with easy production of recombinant proteins, which makes them favorite targets for structural studies and rapid binding screening. For example, ligand screening with OBPs could allow the identification of chemical properties for better binding, such as chain length, molecular volume, functional groups, and bond unsaturation. These, combined with new protein structure prediction methods as used in the design of medical drugs and antibodies, such as homology modeling, dynamics simulations, and molecular docking, could place insect OBPs in a favorite position over ORs as targets for the development of control agents in pest management.

Insect ORs seem more specifically tuned to odorants than OBPs. The higher specificity shown by ORs and the chance of activation/inhibition of specific receptors for a given behavior make these proteins as attractive targets to manipulate pest behaviors. The feasibility of the inhibition of either ORx/ORco complex or ORco by antagonists comprises a promising strategy to disrupt insect specific behavior, such as mating via sex pheromone receptors. However, the lack of structural information is the bottleneck in using insect ORs as targets for semiochemical activity predictions. Tables 1, 2 summarize the number of *OBPs* and *ORs* that have been identified in insect species by transcriptome (i.e., RNA-seq) and genome sequencing. Most of insects studied so far have at least twice *ORs* than *OBPs* according to genome studies. Moreover, there is an extensive expansion of *ORs* in social insects from the Hymenopteran order such as the honey bee *A. mellifera* with 170 *ORs* (Weinstock et al., 2006), and the ants *Solenopsis invicta* and *Cerapachys biroi* with 400 and 506 *ORs*, respectively (Wurm et al., 2011; Oxley et al., 2014). Similarly, the OR expansion is also evident in some agricultural pests, such as the red flour beetle *T. castaneum* with 265 *ORs* compared to 47 *OBPs* (Richards et al., 2008). This makes a demanding task for the target OR selection together with the difficulty for the functional expression of transmembrane proteins such as ORs in order to screen a large number of ligands. An approximation of important properties in both OBPs and ORs are summarized in Table 3.

**Table 3:** Approximate comparison of insect ORs and OBPs according to properties.

**Comparison properties**	**OBPs**	**ORs**
Function	Transport Scavenging Solubilization	Olfactory signal transduction
Sub-classes to focus on	PBPs and GOBPs for Lepidopterans	PRs for Lepidopterans
Molecular characteristics	~18 kDa	~50 kDa
Heterologous expression system	Bacteria (*E. coli*)	*Xenopus laevis* oocytes HEK293 cells *Drosophila*
Binding specificity	Wider range of volatiles	Narrow type of volatiles to very specific in some cases
Structural information	From crystals and NMR Homology modeling	Not available from crystals or NMR yet Homology models proposed
Tissue expression patterns	Mostly antennae Proboscis Female glands	Female and male antennae Female antennae for PRs
Presence across developmental stages	Mainly adult stage	Larvae and adult stages
Application	Semiochemical discovery Biosensors Pollutant scavengers	Semiochemical discovery Receptor blockage

## Further perspectives

The functional characterization of insect ORs as well as their proven roles in insect olfaction have shed lights on the sensitivity and specificity of these insect-specific proteins. These advances will further enhance their feasibility as pest control targets by the understanding of molecular recognition mechanisms and combinatory interactions with OBPs. On the other hand, the current massive effort in the identification and binding characterization of OBPs in several agricultural important insect species will continue and provide more information on their functions in insect physiology. Thus, this review proposes as main advantage for OBPs over ORs, the availability of 3D crystal and NMR structures, which with downstream approaches, such as homology modeling (when necessary), molecular docking and molecular dynamics, would refine the search of bioactive chemicals. This last in complement with ligand affinity measurement will accelerate the study of insect OBPs to be reconsidered as the targets for semiochemical discovery and the tools to design super-ligands in pest control management.

The appearance and development of insecticide resistance in insect pests have led to the intensive research on insect olfaction and the mechanisms that are involved for neural processing. It is well-established that a number of receptors and enzymes in insect CNS are the targets for insecticide resistance development (Figure 1). It has been demonstrated that acetylcholinesterase (AChE) in soluble form provides the resistance to organo-phosphorus and carbamate insecticides, acting as bioscavengers (Lee et al., 2015). Similarly, multiple insecticide resistance mechanisms have been demonstrated in the aphid *M. persicae*, involving carboxylesterases, sodium channels, γ-aminobutyric acid (GABA) and nAChR (Bass et al., 2014). As important components in insect periphery nerve system and key players in insect behaviors, both insect OBPs and ORs represent alternative targets for the identification of compounds with semiochemical activity (or agonist effect) and tools to design strong antagonists to enhance desired behavioral responses of insect pests and reduce the use of insecticides and subsequent resistance.

## Author contributions

HV wrote sections about OBP's structure and ORs, developed tables and figure. J-JZ conceived the idea for the review article, wrote the main section, such as introduction, OBPs function and structure as well as OR-OBP comparison.

### Conflict of interest statement

The authors declare that the research was conducted in the absence of any commercial or financial relationships that could be construed as a potential conflict of interest.
